# Ultraviolet-B Radiation and Nitrogen Affect Nutrient Concentrations and the Amount of Nutrients Acquired by Above-Ground Organs of Maize

**DOI:** 10.1100/2012/608954

**Published:** 2012-05-01

**Authors:** Carlos M. Correia, João F. Coutinho, Eunice A. Bacelar, Berta M. Gonçalves, Lars Olof Björn, José Moutinho Pereira

**Affiliations:** ^1^Department of Biology and Environment, Centre for the Research and Technology of Agro-Environment and Biological Sciences, University of Trás-os-Montes e Alto Douro, 5001-801 Vila Real, Portugal; ^2^Department of Soil Science, Centre of Chemistry, University of Trás-os-Montes e Alto Douro, 5001-801 Vila Real, Portugal; ^3^Key Laboratory of Ecology and Environmental Science in Guangdong Higher Education, School of Life Science, South China Normal University, Guangzhou 510631, China; ^4^Department of Biology, Lund University, 22362 Lund, Sweden

## Abstract

UV-B radiation effects on nutrient concentrations in above-ground organs of maize were investigated at silking and maturity at different levels of applied nitrogen under field conditions. The experiment simulated a 20% stratospheric ozone depletion over Portugal. At silking, UV-B increased N, K, Ca, and Zn concentrations, whereas at maturity Ca, Mg, Zn, and Cu increased and N, P and Mn decreased in some plant organs. Generally, at maturity, N, Ca, Cu, and Mn were lower, while P, K, and Zn concentrations in stems and nitrogen-use efficiency (NUE) were higher in N-starved plants. UV-B and N effects on shoot dry biomass were more pronounced than on nutrient concentrations. Nutrient uptake decreased under high UV-B and increased with increasing N application, mainly at maturity harvest. Significant interactions UV-B x N were observed for NUE and for concentration and mass of some elements. For instance, under enhanced UV-B, N, Cu, Zn, and Mn concentrations decreased in leaves, except on N-stressed plants, whereas they were less affected by N nutrition. In order to minimize nutritional, economical, and environmental negative consequences, fertiliser recommendations based on element concentration or yield goals may need to be adjusted.

## 1. Introduction

Solar UV-B radiation (280–315 nm) levels have changed as a result of stratospheric ozone depletion caused by large-scale emissions of anthropogenic pollutants. Currently, it is estimated that elevated fluxes of UV-B radiation will be a continuing phenomenon until the middle of this century [1].

Although the UV-B radiation comprises only a small part of the solar radiation reaching the surface of the earth, it has a disproportionately large photobiological effect on plants due to its absorption by important molecules, such as proteins, hormones, pigments, and nucleic acids. Plant UV-B research has demonstrated that UV-B radiation has considerable consequences at many levels, including on anatomy, morphology, physiology, biochemistry, phenology, and yield, even though these responses varied markedly within and between species [2, 3].

The sustainability of any crop production depends on maintaining soil plant nutrient levels, mainly nitrogen (N), since it is the nutrient that is most often limiting in agro-ecosystems, resulting in fertilizer N being the most common and often the most expensive fertilizer addition for the production of nonlegume crops [4]. Thus, the study of the interactive effects of enhanced UV-B and N is important because of its potential impact on crop productivity, economic stability of agriculture, and environmental quality.

In view of the worldwide socioeconomic importance of maize (*Zea mays *L.), much research has been conducted on the effects of elevated UV-B radiation (e.g., [5–10]), but only two studies have been done in interaction with N fertilization [11, 12]. Moreover, little is known about UV-B effects on maize plant nutrients. Merely one work investigated the effects of UV-B radiation on iron content and distribution in maize [13]. In the present study maize was grown in field under ambient and enhanced UV-B radiation, at four levels of applied nitrogen with the objective of evaluating the impacts of UV-B and N on nutrient concentrations and the amount of nutrient acquired by above-ground organs at two phenological stages. We hypothesized that UV-B radiation and nitrogen fertilization change the biomass, the nutrient concentrations, and the nutrient uptake of maize and thus fertiliser practice in an enhanced UV-B environment should be adjusted.

## 2. Materials and Methods

### 2.1. Plant Material and Growth Conditions

The experiment was conducted at the University of Trás-os-Montes e Alto Douro, Vila Real, Portugal (41°19′N, 7°44′W). The climate is typical Mediterranean, with mild rainy winters and long, hot, sunny, and dry summers. Mean annual rainfall is about 1100 mm, mainly from October to April. The warmest months are July and August, with mean daily temperatures of 21-22°C. Mean annual sunshine values are 2392 h, with the highest monthly value (342 h) in July. The experimental design was a factorial arrangement in randomized complete blocks with three replicates. Each plot (8.25 m × 2 m) included three “useful lines”, each limited by two border lines. The first “useful line” was consigned to the silking (50% of plants with emerged silks) harvest (9 weeks after emergence), the second to the physiological and biochemical studies [12], and the third to the maturity harvest (16 weeks after emergence). At silking and maturity harvest, the five central plants from the “useful line” were harvested and the dry weights of each above-ground plant organ (after drying in a force-draft oven at 70°C to a constant weight) were evaluated. The treatments consisted of two UV-B radiation levels, high UV-B treatment (UV) and ambient UV-B treatment (C), combined with four nitrogen levels (0 (N0), 100 (N1), 200 (N2), and 300 (N3) kg ha^−1^ of N). Nitrogen fertilizer was applied as urea.

High UV-B treatment was supplied by preburned Philips sun lamps (TL 40W/12) wrapped with 0.1 mm cellulose acetate film (Ultraphan, Weil am Rhein, Germany) and began immediately after the plants emerged. The filters were replaced twice a week to keep uniform optical properties. Lamps were in frames that were adjusted weekly to maintain the UV-B levels on the canopy during the course of the experiment. The experiment simulated a 20% stratospheric ozone reduction in Vila Real (Portugal). Biologically effective UV-B (UV-B_BE_) doses were based on calculations by Björn and Murphy [14] using the generalised plant action spectrum, normalised at 300 nm, in accordance with the mathematical function elaborated by Thimijan et al. [15]. On the summer solstice with clear sky conditions, the supplemental UV-B_BE_ dose was 3.16 KJ m^−2^ day^−1^ in addition to the effective 6.84 kJ m^−2^ day^−1^ UV-B_BE_ from the sky. The homogeneity of the UV-B irradiance from the lamps was measured after sunset (i.e., in the absence of ambient UV-B radiation) with an IL 1400A radiometer (International Light Inc., Newburyport, USA) with a photodetector (SEL 240). The spectral sensitivity of the radiometer and the corresponding correction factor were previously determined with an OL754 spectroradiometer (Optronic, Orlando, USA). The high UV-B treatment was suspended on cloudy days to prevent abnormally high UV-B to photosynthetically active radiation (PAR) ratio. Above the control treatment area, nonburning UV-B frames were used to create shade, as in the UV-B radiated experimental groups. In this way, the visible light environment under control and UV-B frames was similar. Shading from the lamps and lamp supports was estimated with a ceptometer (Decagon Sunfleck Ceptometer, Pullman, WA, USA). During a clear day, with maximum shading (i.e., with low zenith angle), the plant tops received about 90% of the PAR found above the frames. Less shading is expected with increased zenith angle. With this system a small increase in UV-A radiation under the UV-B frames was observed. The daily-integrated percent increase in UV-A was around 2%. However, under the high PAR levels in the field, the additional UV-A irradiances would be considered neutral in effect and their careful control unnecessary [16].

The yellow hybrid DeKalb 502 was used. Triticale was grown on the site until 2 months before sowing to reduce the level of soil-available N. After triticale was removed, the land was prepared by conventional tillage. Based on soil analyses, 90 kg ha^−1^ of P_2_O_5_ as superphosphate (18% P_2_O_5_) and 180 kg ha^−1^ of K_2_O as potassium chloride were applied broadcast and incorporated prior to sowing. Half of the N was applied broadcast before sowing, and the remainder was sidedressed as a band when plants reached a height of 40–50 cm. Maize was oversown at a within-row spacing of 0.15 m spaced 0.75 m apart and thinned to a final density of 9 plants m^−2^. Rainfall was supplemented with furrow irrigation when necessary to ensure that the crop did not suffer water stress. Weeds were controlled manually.

### 2.2. Nutrient Concentration and Amount of Nutrients

After dried, all leaves of five plants per plot were grounded and a sample of the mix was taken for chemical analyses. The same procedure was done for stems and grains. N and P were determined by molecular absorption spectrophotometry (SanPlus, Skalar, The Netherlands), after digestion with H_2_SO_4_ and H_2_O_2_ [17]. Plant concentration of other elements (Ca, Mg, Fe, Cu, Zn, and Mn) was determined by atomic absorption spectrophotometry (3100, Perkin Elmer, USA), and K was determined by flame emission photometry (PFP7, Jenway, UK), after digestion with HNO_3_ and HClO_4_ [17]. The corresponding aboveground biomass data, which were used to calculate the quantity of nutrients accumulated in aboveground plant organs, are presented in Table 1. Growth and yield responses of the plants have been documented elsewhere [11]. Nutrient concentration was calculated on a dry weight basis and the amount of nutrients was expressed per m^2^ of ground area.

Physiological nitrogen-use efficiency (NUE) has been calculated as grain yield per unit N acquired.

### 2.3. Statistical Analysis

All data were subjected to an analysis of variance that tested the UV-B radiation, nitrogen, and UV-B x nitrogen interaction effects with prior data transformation when required. All data sets satisfied the assumptions of ANOVA based on homogeneity of variances, normality of errors, and independence of errors [18]. Significant different means were separated using the Fisher's LSD test.

## 3. Results

### 3.1. UV-B Effects

UV-B radiation had effects on plant nutrient concentrations, although dependent on the phenological phase (Tables 2–10). At silking, enhanced UV-B radiation increased the concentrations of N (both in leaves and stems), and K, Ca, and Zn in stems, whereas P, Mg, Fe, Cu, and Mn were unaffected. Higher heterogeneity was observed at maturity harvest, since UV-B radiation decreased the concentration of N (in stems), P (in all plant organs), and Mn (in leaves and grains) and increased the concentration of Ca and Zn (in stems), Mg (in leaves and stems), and Cu (in grains).

**Figure 1:**
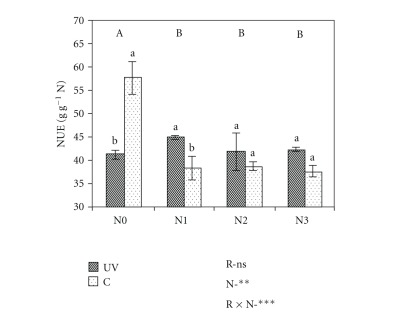
Interactive effects of UV-B radiation and nitrogen on NUE (g g^−1^N) of maize. Means ± S.E. within each N level followed by the same letter (capital letters for N effects) were not significantly different at *P* < 0.10. Significance of main effects: ns: not significant, **P < 0.01, ***P < 0.001; UV: high UV-B treatment; C: ambient UV-B treatment; N0, N1, N2, N3: 0, 100, 200 and 300 kg N ha^−1^.

The total quantity of nutrients present in the crop reflects treatment effects on both tissue concentrations and biomass production (Tables 1–10). At silking harvest, enhanced UV-B decreased the amount of N, P, Ca, and Mg (both in leaves and stems), and K, Fe, and Zn (in leaves), whereas Cu and Mn were not significantly affected. More drastic, effects of UV-B radiation were found at maturity (Tables 2–10). The uptake of all nutrients studied generally decreased under enhanced UV-B radiation, although in few cases significant differences were not reported for specific plant organs. In fact, no statistical differences were found between all treatments for Ca, Fe, Zn, and Mn mass in stems, Mg mass in leaves and stems and Cu mass in grains. A small tendency, although not significant, for higher nitrogen-use efficiency was reported in UV-B treated plants (Figure 1).

### 3.2. Nitrogen Effects

Nitrogen application had small effects on nutrient concentrations and big effects on nutrient acquired at both harvests, although more evident at final than at silking harvest (Tables 2–10). On the other hand, those effects depended on plant organ. At silking, N, Ca, and Zn (in leaves and stems) and Mg, Cu, and Mn (in stems) concentrations were generally lower in N-stressed plants. At maturity harvest, N (in all plant organs), Ca (in leaves and stems) and Cu and Mn (in leaves) concentrations increased with N-supply, while P, K, and Zn (in stems) concentrations were higher in N-starved plants. The mass of the nine elements in all plant organs increased with increasing N application, with few exceptions (Tables 2–10). At silking, only the masses of Mg, Fe, Cu, and Mn in leaves were not significantly different, while at maturity harvest the same occurred to the mass of P, Fe, and Zn in stems and the mass of Fe in leaves. Furthermore, N-stressed plants had the highest NUE (Figure 1).

### 3.3. Interactive Effects

Significant interaction between the UV-B and nitrogen treatments was found in the concentration and amounts of certain elements in specific plant organs, mainly at the maturity harvest (Tables 2–10). At that stage, the concentrations and mass of N, Cu, Zn, and Mn in leaves decreased with UV-B radiation, except on N-starved plants. At the same time, the positive effects of N on these parameters were less evident in UV-B plants. Similar results were verified for the N concentrations in grains, N mass in stems and grains, and Fe mass in grains. Moreover, the UV-B and N effects on K mass in leaves and on P, Ca, Mg, Zn, and Mn mass in grains were inferior at lower N levels and higher UV-B, respectively. In addition, the high NUE in N-starved plants did not occur in an enhanced UV-B environment, while UV-B radiation decreased NUE under N-starved conditions (Figure 1).

## 4. Discussion

### 4.1. UV-B Effects

Changes in nutrient concentrations in plants exposed to supplemental UV-B radiation have been found for some elements in this experiment. For some plant organs, higher concentrations of N, K, Ca, and Zn, at silking, and Ca, Mg, Zn, and Cu, at maturity harvest, in UV-B plants might be associated to a “concentration” effect because of a significant decrease in plant biomass production under enhanced UV-B radiation. However, since there are significant differences among elements, the results indicate that the responses of plant nutrient concentration to UV-B radiation are complex and may also be related to changes in various nutrient metabolic processes. Increases of N concentration in some plant organs and/or plant species were reported by several workers [19–21], while increases of K, Mg, and Zn were found by Yue et al. [22] and an increase of Ca was recorded by Shukla and Kakkar [23]. Meanwhile, the reduction of N, P, and Mn concentrations at maturity harvest, in some plant tissues, suggests a lower absorption capacity after female flowering in UV-B-treated plants, which is reinforced by the lower quantity of nutrients present in the crop, reflecting the concurrent decrease in plant biomass. A decrease of N concentration was verified by He et al. [24], while the drop of P was demonstrated by Musil and Wand [25]. However, as there are no two elements with identical responses to UV-B radiation, optimisation of fertiliser practice in an enhanced UV-B environment would offer a considerable challenge. Several explanations can be given for the reduction of nutrient uptake under high UV-B, including the lower transpiration rate, as reported earlier [12], the inhibition of the activity of nitrate reductase and other key enzymes of nitrogen metabolism [26], the inhibition of ATP synthesis [27], and the decrease of photosynthesis and carbohydrate availability [11]. Despite lower nutrient uptake, these data do not indicate any kind of severe nutrient limitation in high UV-B treated plants.

 As a consequence of lower absorption capacity, principally after female flowering, UV-B-treated plants suffered “self-destruction”, a vicious process of decomposition and remobilization of compounds from vegetative organs to grains, specifically N and P, which increments the senescence and decreases the photosynthetic rate. That aspect is confirmed by nutrient concentrations changes from silking to maturity harvest mainly because of redistribution during grain filling, which in turn is a function of the demand and the sink strength of the grains and of the mobility of the elements within the plants. Earlier senescence and lower photosynthesis promoted by high UV-B were reported previously in maize [6, 11, 12, 28]. Furthermore, the changes in the amount of nutrients acquired by maize shoot under enhanced UV-B will have implications on nutrient cycling within the plant-soil system.

UV-B radiation affected plant biomass quality through changes in the concentration of some elements, namely the decrease of P, Mn, and N and the consequent decrease in crude protein concentration, both in vegetative organs and in grains. Decrease of protein concentration in maize seeds was also reported by Gao et al. [8]. Thus, these changes may be of considerable magnitude at many levels, including in seed germination capacity, nutritional value of food for humans, and animals and plant/herbivore relationships.

Enhanced UV-B had a slight tendency to increase NUE, except on N-starved plants, which suggests a better distribution of nitrogen resources among the different metabolic processes involved in biomass production [29]. The higher NUE first looks advantageous but turns out to be connected with reduction of grain quality, as in other studies [30, 31]. The decrease of NUE by enhanced UV-B in N-stressed treatment was probably related to a higher investment of N in protection mechanisms in these plants. The higher concentration of N, Cu, and Zn and the tendency for higher Mn concentration in leaves of nitrogen-stressed plants jointly with the increment of soluble proteins concentration [12] indicates that N-starved plants exposed to UV-B increased the concentration of those elements necessary for the activation of antioxidant enzymes, such as superoxide dismutase, since Cu, Zn, and Mn are cofactors of such enzymes. Higher concentration and/or activities of antioxidant enzymes were reported in other studies [32, 33]. These responses explain, at least in part, the lower sensibility of N-stressed plants to UV-B radiation, as demonstrated previously [11, 12]. Meanwhile, the absence of UV-B effects on iron concentration, as in silver birch [34], suggests a minor role of this nutrient in maize defence against UV-B radiation, which contrasts to the findings of Zancan et al. [13], who reported increased leaf and root iron contents in maize.

### 4.2. Nitrogen Effects

Nitrogen fertilisation had much less effect on organ nutrient concentration than on the amount of nutrients acquired by above-ground organs reflecting a higher influence of N on plant biomass production. Nonetheless, the decrease of the concentrations of some elements in specific plant organs of N-starved plants was evident, namely, those of N, Ca, Cu, and Mn, although only the N concentration was out of the desired nutrient concentration ranges for maize. This result had significant effects on maize physiology, mainly on carbon metabolism [12], since a greater part of N concentration in leaves is associated with the chloroplasts. The lower amount of nutrients acquired in N-stressed plants, as in other studies [35, 36], namely, for N after silking, indicates the need of decomposition and remobilization of nitrogen compounds accumulated before flowering, a significant fact since the reproductive stage is considered the critical period for N uptake in maize [37]. Furthermore, the continuous absorption of N allows the normal development of embryo, with positive implications on hormonal balance, and the maintenance of enzymatic systems involved in starch and proteins accumulation [38].

 Nitrogen application affects maize grain quality. As in other studies [36, 39], N concentration and thus crude protein concentration were lower in N-stressed plants. Meanwhile, effects of N were not observed on grain concentration of the other elements, which corroborates the findings of Ahmadi et al. [40].

 As expected [35, 36, 41], NUE was higher in N-starved plants, except at high UV-B, for the reasons reported before. Nevertheless, it is possible that the lower NUE in high N doses was also related to higher N losses by NH_3_ volatilization, commonly associated with higher stomatal conductance [42].

## 5. Conclusions

The results of this study indicated the existence of interactive effects between UV-B radiation and nitrogen treatments on nutrient concentration and on the amount of nutrients acquired by maize shoot. In order to minimize nutritional, economical, and environmental negative consequences, fertiliser recommendations for maize based on element concentration in crop shoots or yield goals may need to be adjusted. Moreover, UV-B sensitivity in vegetative organs and grain quality in addition to sensitivity in growth and yield may become important criteria for future plant breeding programs.

## Figures and Tables

**Table 1:** Above-ground shoot dry biomass of maize (g m^−2^) at silking and maturity harvest used to calculate element acquired.

Nitrogen	Radiation	Silking		Maturity		
		Lb	Sb	Lb	Sb	Gb
N0	UV	139.9	287.9 a	161.2 b	563.3	666.9 b
	C	166.1	328.6 a	192.8 a	636.9	854.4 a
	Mean	153.0 C	308.2 C	177.0 D	600.1 C	760.6 D

N1	UV	147.1	341.9 b	197.9 b	682.7	916.1 b
	C	208.3	529.7 a	243.3 a	961.2	1249.9 a
	Mean	177.7 BC	435.8 B	220.6 C	821.9 B	1083.0 C

N2	UV	168.8	385.2 b	206.2 b	752.1	1015.9 b
	C	237.9	633.4 a	296.4 a	1082.8	1499.2 a
	Mean	203.3 B	509.3 A	251.3 B	917.4 B	1257.5 B

N3	UV	197.6	456.5 b	235.6 b	885.3	1125.6 b
	C	275.6	636.9 a	332.3 a	1204.5	1682.8 a
	Mean	236.6 A	546.7 A	283.9 A	1044.9 A	1404.2 A

Mean	UV	163.3 b	367.9 b	200.2 b	720.8 b	931.1 b
	C	222.0 a	532.1 a	266.2 a	971.3 a	1321.5 a

ANOVA						

Radiation (R)		***	***	***	***	***
Nitrogen (N)		***	***	***	***	***
R × N		ns	*	*	ns	*

Lb: leaf biomass; Sb: stem biomass; Gb: grain biomass; UV: high UV-B treatment; C: ambient UV-B treatment; N0, N1, N2, N3: 0, 100, 200, and 300 kg N ha^−1^.

Means within each N level followed by the same letter (capital letters for N effects within each column) were not significantly different at *P* < 0.10. Significance of main effects: ns: not significant; *P < 0.05, ***P < 0.001.

**Table 2:** Interactive effects of UV-B radiation and nitrogen on N concentration (mg g^−1^) and N acquired (g m^−2^) by above-ground organs of maize.

Nitrogen	Radiation	Silking				Maturity					
		Lc	Sc	La	Sa	Lc	Sc	Gc	La	Sa	Ga
N0	UV	33.0	13.4	5.1	4.0	24.7 a	3.4	15.0 a	4.1 a	2.1 a	10.0 a
	C	28.6	10.9	4.8	3.5	17.4 b	3.0	11.3 b	3.4 a	2.0 a	9.5 a
	Mean	30.8 B	12.2 B	4.9 C	3.8 C	21.0 B	3.2 C	13.1 B	3.7 D	2.1 C	9.8 D

N1	UV	38.2	17.0	5.6	5.8	27.0 a	3.8	13.5 b	5.4 b	2.6 b	12.4 b
	C	35.1	14.0	7.3	7.4	29.1 a	5.5	16.5 a	7.1 a	5.2 a	20.6 a
	Mean	36.6 A	15.5 A	6.4 B	6.6 B	28.1 A	4.7 B	15.0 A	6.2 C	3.9 B	16.5 C

N2	UV	38.1	16.5	6.2	6.4	25.8 b	5.1	14.8 a	5.6 b	3.9 b	15.1 b
	C	35.5	15.8	8.4	10.0	30.5 a	6.6	15.1 a	9.0 a	7.1 a	22.6 a
	Mean	36.8 A	16.2 A	7.3 B	8.2 A	28.2 A	5.8 A	15.0 A	7.3 B	5.5 A	18.8 B

N3	UV	39.6	15.8	7.9	7.2	28.0 a	5.2	13.7 b	6.6 b	4.7 b	15.3 b
	C	36.3	14.2	10.0	9.1	29.6 a	5.7	16.7 a	9.8 a	6.9 a	28.1 a
	Mean	37.9 A	15.0 A	8.9 A	8.2 A	28.8 A	5.5 A	15.2 A	8.2 A	5.8 A	21.7 A

Mean	UV	37.5 a	15.8 a	6.3 b	6.0 b	26.6 a	4.4 b	14.1 a	5.5 b	3.4 b	13.4 b
	C	33.9 b	13.7 b	7.6 a	7.5 a	26.7 a	5.2 a	14.9 a	7.3 a	5.3 a	20.2 a

					ANOVA						

Radiation (R)		^+^	*	*	**	ns	*	ns	***	***	***
Nitrogen (N)		*	*	**	***	**	***	^+^	***	***	***
R × N		ns	ns	ns	ns	*	ns	**	**	*	***

Lc: leaf concentration; Sc: stem concentration; La: amount on leaves; Sa: amount on stems; Gc: grain concentration; Ga: amount on grains; UV: high UV-B treatment; C: ambient UV-B treatment; N0, N1, N2, N3: 0, 100, 200, and 300 kg N ha^−1^.

Means within each N level followed by the same letter (capital letters for N effects within each column) were not significantly different at *P* < 0.10. Significance of main effects: ns: not significant; ^+^ *P* < 0.1; *P < 0.05; **P < 0.01; ***P < 0.001.

**Table 3:** Interactive effects of UV-B radiation and nitrogen on P concentration (mg g^−1^) and P acquired (g m^−2^) by above-ground organs of maize.

Nitrogen	Radiation	Silking				Maturity					
		Lc	Sc	La	Sa	Lc	Sc	Gc	La	Sa	Ga
N0	UV	3.19	2.87	0.48	0.83	2.56	0.99 b	3.27	0.42	0.62	2.19 a
	C	3.23	2.73	0.54	0.88	2.72	1.87 a	3.05	0.53	1.18	2.60 a
	Mean	3.21	2.80	0.51 C	0.85 C	2.64	1.43 A	3.16	0.48 C	0.90	2.39 D

N1	UV	3.49	2.77	0.51	0.95	2.52	0.86 b	3.20	0.50	0.58	2.92 b
	C	3.33	2.82	0.69	1.50	2.80	1.19 a	3.39	0.68	1.13	4.24 a
	Mean	3.41	2.80	0.60 BC	1.22 B	2.66	1.02 B	3.30	0.59 BC	0.85	3.58 C

N2	UV	3.38	3.05	0.55	1.18	2.27	1.00 a	3.07	0.50	0.77	3.12 b
	C	3.39	2.85	0.80	1.81	2.93	1.22 a	3.59	0.87	1.31	5.36 a
	Mean	3.38	2.95	0.68 AB	1.49 A	2.60	1.11 B	3.33	0.68 B	1.04	4.24 B

N3	UV	3.38	2.90	0.67	1.33	2.52	0.95 a	3.06	0.59	0.84	3.42 b
	C	3.22	2.46	0.89	1.56	3.15	1.02 a	3.61	1.05	1.22	6.05 a
	Mean	3.30	2.68	0.78 A	1.44 A	2.84	0.99 B	3.33	0.82 A	1.03	4.73 A

Mean	UV	3.37	2.88	0.56 b	1.09 b	2.48 b	0.94 b	3.15 b	0.51 b	0.70 b	2.97 b
	C	3.29	2.71	0.73 a	1.44 a	2.90 a	1.33 a	3.41 a	0.78 a	1.21 a	4.57 a

					ANOVA						

Radiation (R)		ns	ns	***	***	*	**	^+^	***	***	***
Nitrogen (N)		ns	ns	***	***	ns	^+^	ns	**	ns	***
R × N		ns	ns	ns	ns	ns	^+^	ns	ns	ns	***

Lc: leaf concentration; Sc: stem concentration; La: amount on leaves; Sa: amount on stems; Gc: grain concentration; Ga: amount on grains; UV: high UV-B treatment; C: ambient UV-B treatment; N0, N1, N2, N3: 0, 100, 200, and 300 kg N ha^−1^.

Means within each N level followed by the same letter (capital letters for N effects within each column) were not significantly different at *P* < 0.10. Significance of main effects: ns: not significant; ^+^ *P* < 0.1; *P < 0.05; **P < 0.01; ***P < 0.001.

**Table 4:** Interactive effects of UV-B radiation and nitrogen on K concentration (mg g^−1^) and K acquired (g m^−2^) by above-ground organs of maize.

Nitrogen	Radiation	Silking				Maturity					
		Lc	Sc	La	Sa	Lc	Sc	Gc	La	Sa	Ga
N0	UV	21.6	18.0	3.4	5.5	23.2	12.6 a	6.8	3.8 a	8.1	4.5
	C	24.0	20.0	4.0	6.5	22.7	14.7 a	6.4	4.4 a	9.2	5.4
	Mean	22.8	19.0	3.7 B	6.0 C	22.9	13.6 A	6.6	4.1 C	8.6 B	5.0 C

N1	UV	26.7	30.4	3.9	10.1	20.5	10.5 a	6.7	4.0 a	7.2	6.1
	C	18.9	14.4	3.9	7.6	18.7	9.1 a	6.5	4.5 a	8.7	8.2
	Mean	22.8	22.4	3.9 B	8.9 BC	19.6	9.8 B	6.6	4.3 C	8.0 B	7.1 B

N2	UV	22.4	28.0	3.7	10.7	18.4	8.2 b	6.0	4.0 b	6.1	6.2
	C	21.8	19.2	5.1	12.2	20.4	11.7 a	6.7	6.1 a	12.8	10.0
	Mean	22.1	23.6	4.4 AB	11.4 AB	19.4	10.0 B	6.3	5.1 B	9.4 AB	8.1AB

N3	UV	23.7	27.7	4.7	12.9	19.6	13.3 a	6.1	4.6 b	12.2	6.9
	C	22.4	20.5	6.2	13.1	21.6	10.4 a	6.0	7.1 a	12.5	10.1
	Mean	23.1	24.1	5.4 A	13.0 A	20.6	11.9 AB	6.1	5.8 A	12.4 A	8.5 A

Mean	UV	23.9	26.6 a	4.0 b	10.2	20.4	11.3 a	6.4	4.1 b	8.7 b	6.0 b
	C	21.8	18.5 b	4.8 a	9.9	20.8	11.5 a	6.4	5.5 a	10.8 a	8.5 a

					ANOVA						

Radiation (R)		ns	*	^+^	ns	ns	ns	ns	***	^+^	***
Nitrogen (N)		ns	ns	^+^	**	ns	*	ns	**	^+^	***
R × N		ns	ns	ns	ns	ns	^+^	ns	*	ns	ns

Lc: leaf concentration; Sc: stem concentration; La: amount on leaves; Sa: amount on stems; Gc: grain concentration; Ga: amount on grains; UV: high UV-B treatment; C: ambient UV-B treatment; N0, N1, N2, N3: 0, 100, 200, and 300 kg N ha^−1^.

Means within each N level followed by the same letter (capital letters for N effects within each column) were not significantly different at *P* < 0.10. Significance of main effects: ns: not significant; ^+^ *P* < 0.1; *P < 0.05; **P < 0.01; ***P < 0.001.

**Table 5:** Interactive effects of UV-B radiation and nitrogen on Ca concentration (mg g^−1^) and Ca acquired (g m^−2^) by above-ground organs of maize.

Nitrogen	Radiation	Silking				Maturity					
		Lc	Sc	La	Sa	Lc	Sc	Gc	La	Sa	Ga
N0	UV	7.56	4.02	1.10	1.19	8.9	3.04	0.12	1.47	1.92	0.08 a
	C	7.80	3.95	1.31	1.31	8.6	2.21	0.11	1.66	1.40	0.10 a
	Mean	7.68 C	3.98 B	1.21 B	1.25 C	8.8 B	2.63 B	0.12	1.56 C	1.66 C	0.09 C

N1	UV	8.97	5.47	1.30	1.86	9.8	2.73	0.12	1.94	1.86	0.11 a
	C	10.10	3.87	2.10	2.05	11.0	2.73	0.11	2.69	2.64	0.13 a
	Mean	9.53 A	4.67 A	1.70 A	1.95 B	10.4 A	2.73 B	0.11	2.31 B	2.25 B	0.12 B

N2	UV	9.26	4.20	1.53	1.61	11.6	3.78	0.11	2.53	2.86	0.11 b
	C	8.64	3.56	2.05	2.26	10.6	2.89	0.11	3.13	3.12	0.17 a
	Mean	8.95 AB	3.88 B	1.79 A	1.93 B	11.1 A	3.34 A	0.11	2.83 A	2.99 A	0.14 B

N3	UV	8.84	4.89	1.74	2.24	10.4	3.48	0.11	2.43	3.11	0.13 b
	C	7.41	4.41	2.04	2.81	10.5	3.00	0.13	3.47	3.61	0.23 a
	Mean	8.13 BC	4.65 A	1.89 A	2.53 A	10.5 A	3.24 A	0.12	2.95 A	3.36 A	0.18 A

Mean	UV	8.71	4.75 a	1.44 b	1.79 b	10.2	3.23 a	0.12	2.12 b	2.44	0.11 b
	C	8.48	3.95 b	1.88 a	2.10 a	10.2	2.71 b	0.12	2.74 a	2.69	0.16 a

					ANOVA						

Radiation (R)		ns	*	**	*	ns	**	ns	***	ns	***
Nitrogen (N)		^+^	^+^	**	***	*	*	ns	***	***	***
R × N		ns	ns	ns	ns	ns	ns	ns	ns	ns	^+^

Lc: leaf concentration; Sc: stem concentration; La: amount on leaves; Sa: amount on stems; Gc: grain concentration; Ga: amount on grains; UV: high UV-B treatment; C: ambient UV-B treatment; N0, N1, N2, N3: 0, 100, 200, and 300 kg N ha^−1^.

Means within each N level followed by the same letter (capital letters for N effects within each column) were not significantly different at *P* < 0.10. Significance of main effects: ns: not significant, ^+^ *P* < 0.1, *P < 0.05, **P < 0.01, ***P < 0.001.

**Table 6:** Interactive effects of UV-B radiation and nitrogen on Mg concentration (mg g^−1^) and Mg acquired (g m^−2^) by above-ground organs of maize.

Nitrogen	Radiation	Silking				Maturity					
		Lc	Sc	La	Sa	Lc	Sc	Gc	La	Sa	Ga
N0	UV	2.40	2.80	0.35	0.82	3.00	2.08 a	1.22	0.49	1.30	0.82 a
	C	3.24	3.17	0.53	1.05	3.03	2.05 a	1.03	0.58	1.32	0.88 a
	Mean	2.82	2.99 B	0.44	0.94 C	3.01	2.07	1.12	0.53 B	1.31 C	0.85 C

N1	UV	3.23	3.51	0.47	1.19	3.13	2.03 a	1.25	0.62	1.37	1.15 b
	C	4.29	3.51	0.90	1.87	3.05	1.75 a	1.12	0.75	1.68	1.39 a
	Mean	3.76	3.51 A	0.68	1.53 B	3.09	1.89	1.19	0.68 AB	1.53 BC	1.27 B

N2	UV	2.64	2.78	0.44	1.07	3.32	2.42 a	0.96	0.73	1.84	0.98 b
	C	3.09	2.83	0.74	1.79	2.55	1.49 b	1.21	0.76	1.61	1.78 a
	Mean	2.86	2.80 B	0.59	1.43 B	2.93	1.96	1.09	0.74 A	1.73 AB	1.38 B

N3	UV	2.64	3.20	0.51	1.47	3.17	2.08 a	1.17	0.74	1.84	1.30 b
	C	2.67	3.47	0.73	2.21	2.37	1.51 b	1.28	0.79	1.81	2.15 a
	Mean	2.65	3.33 A	0.62	1.84 A	2.77	1.79	1.23	0.77 A	1.82 A	1.73 A

Mean	UV	2.77	3.13	0.45 b	1.17 b	3.16 a	2.13 a	1.16	0.65	1.59	1.10 b
	C	3.32	3.24	0.73 a	1.72 a	2.75 b	1.70 b	1.16	0.72	1.60	1.56 a

						ANOVA					

Radiation (R)		ns	ns	**	***	^+^	**	ns	ns	ns	***
Nitrogen (N)		ns	^+^	ns	**	ns	ns	ns	^+^	^+^	***
R × N		ns	ns	ns	ns	ns	^+^	ns	ns	ns	**

Lc: leaf concentration; Sc: stem concentration; La: amount on leaves; Sa: amount on stems; Gc: grain concentration; Ga: amount on grains; UV: high UV-B treatment; C: ambient UV-B treatment; N0, N1, N2, N3: 0, 100, 200, and 300 kg N ha^−1^.

Means within each N level followed by the same letter (capital letters for N effects within each column) were not significantly different at *P* < 0.10. Significance of main effects: ns: not significant, ^+^ *P* < 0.1, **P < 0.01, ***P < 0.001.

**Table 7:** Interactive effects of UV-B radiation and nitrogen on Fe concentration (*μ*g g^−1^) and Fe acquired (mg m^−2^) by above-ground organs of maize.

Nitrogen	Radiation	Silking				Maturity					
		Lc	Sc	La	Sa	Lc	Sc	Gc	La	Sa	Ga
N0	UV	321.5	51.5 a	49.6	14.8 a	402.0	182.0	28.5	66.4	112.2	19.1 a
	C	362.0	70.0 a	60.8	23.8 a	1029.3	300.0	18.3	204.7	184.8	15.6 a
	Mean	341.8	60.8	55.2	19.3 B	715.6	241.0	23.4	135.5	148.5	17.4 C

N1	UV	367.0	102.0 a	54.8	35.4 a	363.3	228.0	25.7	70.5	155.1	23.4 a
	C	360.7	58.3 b	75.2	31.0 a	445.0	154.3	20.7	108.9	150.5	25.7 a
	Mean	363.8	80.2	65.0	33.2 A	404.2	191.2	23.2	89.7	152.8	24.6 B

N2	UV	301.5	59.5 b	50.2	23.2 b	392.5	315.5	29.5	86.3	238.3	29.9 b
	C	397.3	101.3 a	95.0	64.4 a	448.7	157.7	29.3	133.8	171.5	43.1 a
	Mean	349.4	80.4	72.6	43.8 A	420.6	236.6	29.4	110.1	204.9	36.5 A

N3	UV	375.0	84.7 a	73.1	38.8 a	504.3	281.7	26.0	119.1	231.3	28.9 b
	C	289.3	48.0 b	79.7	30.6 a	471.0	194.3	33.0	156.1	230.8	55.1 a
	Mean	332.2	66.3	76.4	34.7 A	487.7	238.0	29.5	137.6	231.0	42.0 A

Mean	UV	347.2	78.2	58.3 b	29.9	419.2	252.4	27.1	87.4 b	186.0	25.5 b
	C	352.3	69.4	77.7 a	37.4	598.5	201.6	25.3	150.9 a	184.4	34.9 a

					ANOVA						

Radiation (R)		ns	ns	*	ns	ns	ns	ns	*	ns	**
Nitrogen (N)		ns	ns	ns	^+^	ns	ns	ns	ns	ns	***
R × N		ns	*	ns	*	ns	ns	ns	ns	ns	*

Lc: leaf concentration; Sc: stem concentration; La: amount on leaves; Sa: amount on stems; Gc: grain concentration; Ga: amount on grains; UV: high UV-B treatment; C: ambient UV-B treatment; N0, N1, N2, N3: 0, 100, 200, and 300 kg N ha^−1^.

Means within each N level followed by the same letter (capital letters for N effects within each column) were not significantly different at *P* < 0.10. Significance of main effects: ns: not significant, ^+^ *P* < 0.1, *P < 0.05, **P < 0.01, ***P < 0.001.

**Table 8:** Interactive effects of UV-B radiation and nitrogen on Cu concentration (*μ*g g^−1^) and Cu acquired (mg m^−2^) by above-ground organs of maize.

Nitrogen	Radiation	Silking				Maturity					
		Lc	Sc	La	Sa	Lc	Sc	Gc	La	Sa	Ga
N0	UV	11.50	2.00	1.79	0.57	17.5 a	6.00	2.00	2.88 a	3.78	1.34
	C	9.67	3.00	1.59	0.98	11.7 b	6.00	1.33	2.26 a	3.58	1.14
	Mean	10.59	2.50 B	1.69	0.78 B	14.6 B	6.00	1.67	2.57 C	3.68 B	1.24 B

N1	UV	14.00	3.33	2.06	1.12	14.3 b	3.67	1.67	2.84 b	2.58	1.55
	C	14.33	3.00	2.99	1.60	19.0 a	5.33	1.33	4.63 a	5.19	1.66
	Mean	14.17	3.16 B	2.52	1.36 B	16.7 B	4.50	1.50	3.73 B	3.89 B	1.60 B

N2	UV	12.00	3.50	2.00	1.37	18.5 b	5.00	1.50	4.05 b	3.77	1.52
	C	11.00	3.67	2.55	2.32	24.3 a	5.00	1.33	7.20 a	5.32	1.95
	Mean	11.50	3.59 AB	2.27	1.85 AB	21.4 A	5.00	1.42	5.63 A	4.54 AB	1.73 B

N3	UV	14.33	7.00	2.94	3.25	22.0 a	5.33	2.33	5.10 b	4.79	2.67
	C	10.67	4.00	2.92	2.54	22.7 a	5.67	1.67	7.53 a	6.84	2.78
	Mean	12.50	5.50 A	2.93	2.90 A	22.3 A	5.50	2.00	6.32 A	5.81 A	2.72 A

Mean	UV	13.20	4.20	2.26	1.70	18.1	4.90	1.90 a	3.77 b	3.72 b	1.84
	C	11.42	3.42	2.51	1.86	19.4	5.50	1.42 b	5.41 a	5.23 a	1.88

					ANOVA						

Radiation (R)		ns	ns	ns	ns	ns	ns	^+^	***	*	ns
Nitrogen (N)		ns	^+^	ns	*	**	ns	ns	***	^+^	*
R × N		ns	ns	ns	ns	*	ns	ns	**	ns	ns

Lc: leaf concentration; Sc: stem concentration; La: amount on leaves; Sa: amount on stems; Gc: grain concentration; Ga: amount on grains; UV: high UV-B treatment; C: ambient UV-B treatment; N0, N1, N2, N3: 0, 100, 200, and 300 kg N ha^−1^.

Means within each N level followed by the same letter (capital letters for N effects within each column) were not significantly different at *P* < 0.10. Significance of main effects: ns: not significant, ^+^ *P* < 0.1, *P < 0.05, **P < 0.01, ***P < 0.001.

**Table 9:** Interactive effects of UV-B radiation and nitrogen on Zn concentration (*μ*g g^−1^) and Zn acquired (mg m^−2^) by above-ground organs of maize.

Nitrogen	Radiation	Silking				Maturity					
		Lc	Sc	La	Sa	Lc	Sc	Gc	La	Sa	Ga
N0	UV	24.0 a	25.5 a	3.8	7.6 a	97.0 a	66.0	26.5	16.0 a	39.4	17.9 a
	C	31.3 a	32.0 a	5.2	10.1 a	40.3 b	33.3	22.7	7.9 b	21.1	19.3 a
	Mean	27.6 C	28.8 B	4.5 C	8.9 C	68.7	49.7 A	24.6	11.9 C	30.3	18.6 D

N1	UV	35.3 a	47.0 a	5.2	15.7 a	67.3 a	40.7	26.3	13.5 a	27.3	23.9 a
	C	39.7 a	35.0 a	8.3	18.5 a	81.7 a	24.0	23.3	19.8 a	23.2	29.1 a
	Mean	37.5 B	41.0 A	6.7 B	17.2 B	74.5	32.3 B	24.8	16.6 C	25.3	26.5 C

N2	UV	39.5 a	45.5 a	6.5	17.5 b	63.5 b	28.0	24.0	13.9 b	21.1	24.5 b
	C	51.3 a	47.7 a	12.1	30.2 a	100.3 a	26.3	30.7	29.6 a	29.4	45.4 a
	Mean	45.4 AB	46.6 A	9.3 A	23.9 A	81.9	27.2 B	27.3	21.7 B	25.3	34.9 B

N3	UV	58.7 a	61.3 a	11.3	28.4 a	86.7 a	28.3	26.0	19.9 b	24.8	29.0 b
	C	39.7 b	34.0 b	10.9	21.7 a	105.7 a	28.3	32.3	35.0 a	34.0	54.1 a
	Mean	49.2 A	47.7 A	11.1 A	25.0 A	96.2	28.3 B	29.2	27.5 A	29.4	41.5 A

Mean	UV	40.9	46.7 a	7.0 b	18.3	78.3	39.5 a	25.8	16.0 b	27.8	24.3 b
	C	40.5	37.2 b	9.1 a	20.2	82.0	28.0 b	27.3	23.1 a	26.9	37.0 a

					ANOVA						

Radiation (R)		ns	^+^	*	ns	ns	^+^	ns	**	ns	***
Nitrogen (N)		*	*	***	***	ns	^+^	ns	***	ns	***
R × N		^+^	*	ns	^+^	*	ns	ns	**	ns	**

Lc: leaf concentration; Sc: stem concentration; La: amount on leaves; Sa: amount on stems; Gc: grain concentration; Ga: amount on grains; UV: high UV-B treatment; C: ambient UV-B treatment; N0, N1, N2, N3: 0, 100, 200, and 300 kg N ha^−1^.

Means within each N level followed by the same letter (capital letters for N effects within each column) were not significantly different at *P* < 0.10. Significance of main effects: ns: not significant, ^+^ *P* < 0.1, *P < 0.05, **P < 0.01, ***P < 0.001.

**Table 10:** Interactive effects of UV-B radiation and nitrogen on Mn concentration (*μ*g g^−1^) and Mn acquired (mg m^−2^) by above-ground organs of maize.

Nitrogen	Radiation	Silking				Maturity					
		Lc	Sc	La	Sa	Lc	Sc	Gc	La	Sa	Ga
N0	UV	51.5	35.0	7.7	10.6	119.5 a	59.5	7.5	19.7 a	37.2	5.0 a
	C	53.7	42.7	8.8	13.8	97.0 a	48.7	7.7	18.7 a	31.0	6.5 a
	Mean	52.6	38.8 B	8.2	12.2 C	108.2 C	54.1	7.6	19.2 D	34.1 C	5.8 B

N1	UV	95.7	49.7	14.2	17.1	155.7 a	66.7	9.0	31.0 b	44.7	8.2 b
	C	73.7	48.0	15.4	25.3	181.0 a	56.3	9.7	44.4 a	54.5	12.0 a
	Mean	84.7	48.8 AB	14.8	21.2 B	168.3 B	61.5	9.3	37.7 C	49.6 BC	10.1 A

N2	UV	67.5	56.5	11.1	21.7	168.5 b	65.5	6.5	37.2 b	48.7	6.6 b
	C	105.3	63.7	24.4	40.6	265.7 a	52.3	8.7	78.7 a	57.9	12.9 a
	Mean	86.4	60.1 A	17.8	31.1 A	217.1 A	58.9	7.6	57.9 B	53.3 AB	9.8 A

N3	UV	104.7	74.7	20.5	34.8	164.3 b	59.7	6.7	38.5 b	53.9	7.5 b
	C	77.0	47.7	21.2	30.4	342.0 a	66.7	8.7	113.2 a	80.6	14.6 a
	Mean	90.8	61.2 A	20.9	32.5 A	253.2 A	63.2	7.7	75.9 A	67.2 A	11.1 A

Mean	UV	83.9	55.6	14.2	22.0	153.6 b	62.9	7.5 b	32.2 b	46.7	7.1 b
	C	77.4	50.5	17.5	27.5	221.4 a	56.0	8.7 a	63.8 a	56.0	11.5 a

					ANOVA						

Radiation (R)		ns	ns	ns	ns	***	ns	^+^	***	ns	***
Nitrogen (N)		ns	^+^	ns	**	***	ns	ns	***	*	**
R × N		ns	ns	ns	ns	**	ns	ns	***	ns	^+^

Lc: leaf concentration; Sc: stem concentration; La: amount on leaves; Sa: amount on stems; Gc: grain concentration; Ga: amount on grains; UV: high UV-B treatment; C: ambient UV-B treatment; N0, N1, N2, N3: 0, 100, 200, and 300 kg N ha^−1^.

Means within each N level followed by the same letter (capital letters for N effects within each column) were not significantly different at *P* < 0.10. Significance of main effects: ns: not significant, ^+^ *P* < 0.1, *P < 0.05, **P < 0.01, ***P < 0.001.
